# Reanalysis of *BRCA1/2* negative high risk ovarian cancer patients reveals novel germline risk loci and insights into missing heritability

**DOI:** 10.1371/journal.pone.0178450

**Published:** 2017-06-07

**Authors:** Jaime L. Stafford, Gregory Dyson, Nancy K. Levin, Sophia Chaudhry, Rita Rosati, Hasini Kalpage, Courtney Wernette, Nancie Petrucelli, Michael S. Simon, Michael A. Tainsky

**Affiliations:** 1 Center for Molecular Medicine and Genetics, Wayne State University School of Medicine, Detroit, MI, United States of America; 2 Department of Oncology, Wayne State University School of Medicine, Detroit, MI, United States of America; 3 Molecular Therapeutics Program, Karmanos Cancer Institute at Wayne State University School of Medicine, Detroit, MI, United States of America; Ohio State University Wexner Medical Center, UNITED STATES

## Abstract

While up to 25% of ovarian cancer (OVCA) cases are thought to be due to inherited factors, the majority of genetic risk remains unexplained. To address this gap, we sought to identify previously undescribed OVCA risk variants through the whole exome sequencing (WES) and candidate gene analysis of 48 women with ovarian cancer and selected for high risk of genetic inheritance, yet negative for any known pathogenic variants in either *BRCA1* or *BRCA2*. *In silico* SNP analysis was employed to identify suspect variants followed by validation using Sanger DNA sequencing. We identified five pathogenic variants in our sample, four of which are in two genes featured on current multi-gene panels; (*RAD51D*, *ATM*). In addition, we found a pathogenic *FANCM* variant (R1931*) which has been recently implicated in familial breast cancer risk. Numerous rare and predicted to be damaging variants of unknown significance were detected in genes on current commercial testing panels, most prominently in *ATM* (n = 6) and *PALB2* (n = 5). The *BRCA2* variant p.K3326*, resulting in a 93 amino acid truncation, was overrepresented in our sample (odds ratio = 4.95, p = 0.01) and coexisted in the germline of these women with other deleterious variants, suggesting a possible role as a modifier of genetic penetrance. Furthermore, we detected loss of function variants in non-panel genes involved in OVCA relevant pathways; DNA repair and cell cycle control, including *CHEK1*, *TP53I3*, *REC8*, *HMMR*, *RAD52*, *RAD1*, *POLK*, *POLQ*, and *MCM4*. In summary, our study implicates novel risk loci as well as highlights the clinical utility for retesting *BRCA1/2* negative OVCA patients by genomic sequencing and analysis of genes in relevant pathways.

## Introduction

Recent studies suggest that up to 25% of epithelial ovarian cancer cases arise due to an inherited risk factor.[1][2] Hereditary breast and ovarian cancer (HBOC) syndromes are, for the most part, autosomal dominant genetic disorders in which germline mutations elevate lifetime risk of developing breast or ovarian cancer up to as much as 80% and 39%, respectively[3]. The risk of among the general population is 12% for breast and 1.4% for ovarian cancer[4]. Therefore, women with a personal or family history of OVCA and/or young onset and/or multiple cases of breast cancer are counseled to consider genetic screening per guidelines of the National Comprehensive Cancer Network (NCCN) (Genetic/Familial High-Risk Assessment: Breast and Ovarian www.nccn.org). Current testing panels mostly feature genes involved in DNA repair and cell cycle control, such as *BRCA1* and *BRCA2*, which explain the majority of inherited ovarian and breast cancer, as well as 22 other genes including *TP53*, *PTEN*, *CDH1*, *ATM*, *CHEK2*, *PALB2*, and mismatch repair genes, *MSH1*, *MSH2*

High-throughput sequencing using next generation technology allows for a more efficient and unbiased approach in the discovery of novel cancer predisposition loci and has helped to determine the frequency of germline mutations in HBOC. However, study participants are rarely selected based on family history, meaning that most of the underlying etiology is sporadic, and the majority of the causal variants uncovered are in *BRCA1* and *BRCA2*, genes that already have well established roles in OVCA. The purpose of our study was to address the issue of missing heritability in ovarian cancer by focusing on 48 women with a personal history of OVCA and who were at high risk of genetic inheritance but had no known pathogenic variant in *BRCA1/BRCA2*. Whole exome sequencing followed by current panel and candidate gene analysis identified four pathogenic germline mutations in current HBOC panel genes; *ATM* and *RAD51D*, two of which are novel and not normally associated with OVCA. Additionally, a nonsense variant considered pathogenic in *FANCM*, a gene not featured on HBOC testing panels was uncovered.

## Results

### Clinically actionable variants in sample

We performed WES on blood DNA from 48 women with a personal history of OVCA and determined to be at high risk for inheritance of a germline predisposition mutation, but with no known deleterious mutations in *BRCA1/BRCA2* (Table 1). We identified a total of 5 pathogenic loss of function variants. (Table 2.1) Four of which were in genes currently featured on newer comprehensive HBOC panels; two novel frameshift variants in *ATM* (c.2503_2507del and c.5697_5698insA) and two truncating variants in *RAD51D (*rs587781756 p.Q171* and rs387906843 p.R206*, as well as a pathogenic variant in a non-panel gene, *FANCM* (rs144567652 p.R1931*) previously found to be strongly associated with hereditary risk of breast cancer[5]. *ATM* (Ataxia Telangiectasia Mutated) codes for a protein kinase important for DNA damage recognition and activation of substrates including p53, BRCA1, and other homologous recombination repair factors. Homozygous mutations in *ATM* cause ataxia-telangiectasia, a rare inherited autosomal recessive disorder which affects the immune and nervous system, and leads to increased sensitivity to radiation. Although heterozygous *ATM* mutation carriers do not have ataxia-telangiectasia, they have a 17–52% lifetime risk of developing breast cancer.[6] However, despite association of *ATM* with ovarian cancer in recent literature[7], carriers are not routinely counseled for this risk as exact risks are unknown. One patient with an *ATM* pathogenic variant in our sample (OCF28-1) had a family history of liver, lung (n = 2) and breast cancer, on the same parental side of the family. The proband herself was first diagnosed with breast cancer at the age of 48 before a secondary diagnosis of OVCA at 57. The second carrier of an *ATM* frameshift mutation (OCL56) was diagnosed at 73, and had a family history of OVCA (two additional cases besides herself) as well as two cases of breast cancer, all on the maternal side.

**Table 1:** Characteristics of ovarian cancer subjects (*N* = 48).

Mean Age at Diagnosis	52.8 (yrs)	25–81 (range)
**Histology**	**n** =	**%**
Serous	26	54
Endometrioid	5	10
Mixed	4	8
Adenocarcinoma, NOS	2	4
Clear Cell	1	2
Mucinous	1	2
Unknown	9	19
**Stage**	**n** =	**%**
I	8	17
II	5	10
III	23	48
IV	3	6
Unknown	9	19
**Grade**	**n** =	**%**
Grade 1- well differentiated	1	2
Grade 2- moderate	6	13
Grade 3- poor	24	50
Unknown	17	35
**Personal and Family History**	**n** =	**%**
personal BC/OVCA diagnosis < 50 yrs of age	15	31
personal second primary cancer diagnosis	12	25
personal/family history of BC	31	65
family history of OVCA	14	29
family history of epithelial cancer	47	98

**Table 2:** Variants in sample in genes associated with breast or ovarian cancer.

**Table 2.1 Clinically Actionable Variants**														
**ID**	**Gene**	**Consequence**	**AA**	**dbSNP ID**	** Variant**	**MAF**	**OBS**	**HGMD**						
OCF28-1	ATM	FRAMESHIFT	CATCTG>C	N/A	c.2503_2507del	N/A	1	N/A						
OCL56	ATM	FRAMESHIFT	G>GA	N/A	c.5697_5698insA	N/A	1	N/A						
OCJ19	FANCM	STOP	R1931*	rs144567652	c.5713C>T	0.0009	1	DM						
OCH26	RAD51D	STOP	Q171*	rs587781756	c.511C>T	N/A	1	N/A						
OCK1	RAD51D	STOP	R206*	rs387906843	c.616C>T	0.0001	1	DM						
**Table 2.2 Variants of Unknown Significance in Panel Genes**														
**ID**	**Gene**	**Consequence**	**AA**	**dbSNP ID**	**Variant**	**MAF**	**OBS**	**SIFT**	**PolyPhen**	**MUT PRED**	**LRT**	**MUT TAST**	**GERP**	**HGMD**
OCD13	ATM	MISSENSE	S49C	rs1800054	c.146C>G	0.011	1	DEL	Possibly Damaging	N/A	NEUT	DC	4.22	DM?
OCD16	ATM	MISSENSE	F1463C	rs138327406	c.4388T>G	0.002	3	TOL	Probably Damaging	MEDIUM	DEL	DC	5.56	DM
OCG29	ATM	MISSENSE	F1463C	rs138327406	c.4388T>G	0.002	3	DEL	Probably Damaging	MEDIUM	DEL	DC	5.56	DM
OCK1	ATM	MISSENSE	F1463C	rs138327406	c.4388T>G	0.002	3	DEL	Probably Damaging	MEDIUM	DEL	DC	5.56	DM
OCM13	ATM	MISSENSE	S333F	rs28904919	c.998C>T	0.004	1	DEL	Benign	N/A	NEUT	PM	3.85	DM?
OCJ10	ATM	MISSENSE	N1853V	rs1801673	c.5558A>T	0.006	1	N/A	Benign	MEDIUM	DEL	DC	5.53	DP
OCP43	ATM	MISSENSE	L2307F	rs56009889	c.6919C>T	0.0019	1	DEL	Probably Damaging	N/A	DEL	PM	5.58	DM?
OCP43	ATM	MISSENSE	V2540I	rs35203200	c.7618G>A	0.00005	1	N/A	N/A	N/A	N/A	N/A	N/A	N/A
OCL56	BRCA1	MISSENSE	S1040N	rs4986852	c.3119G>A	0.019	2	TOL	Possibly Damaging	N/A	NEUT	PM	2.01	DM?
OCN45	BRCA1	MISSENSE	S1040N	rs4986852	c.3119G>A	0.019	2	N/A	Possibly Damaging	N/A	NEUT	PM	2.01	DM?
OCD16	BRCA1	MISSENSE	S1533I	rs1800744	c.4535G>T	0.003	1	N/A	Benign	N/A	NEUT	PM	3.98	DM?
OCD16	BRCA2	MISSENSE	T630I	rs80358479	c.1889C>T	0.001	1	DEL	Benign	N/A	N/A	N/A	N/A	N/A
OCN45	BRCA2	MISSENSE	A2717S	rs28897747	c.8149G>T	0.001	1	TOL	Probably Damaging	MEDIUM	DEL	PM	4.5	DM?
OCP9	BRCA2	MISSENSE	E2856A	rs11571747	c.8567A>C	0.001	1	TOL	Probably Damaging	N/A	NEUT	PM	5.28	DM?
OCF28-1	BRCA2	STOP	K3326*	rs11571833	c.9976A>T	0.009	4	N/A	N/A	N/A	N/A	N/A	N/A	DP
OCK1	BRCA2	STOP	K3326*	rs11571833	c.9976A>T	0.009	4	N/A	N/A	N/A	N/A	N/A	N/A	DP
OCN22	BRCA2	STOP	K3326*	rs11571833	c.9976A>T	0.009	4	N/A	N/A	N/A	N/A	N/A	N/A	DP
OCP36	BRCA2	STOP	K3326*	rs11571833	c.9976A>T	0.009	4	N/A	N/A	N/A	N/A	N/A	N/A	DP
OCE27	CHEK2	MISSENSE	I232V	rs587780185	c.565A>G	0.00001	1	DEL	Probably Damaging	N/A	N/A	N/A	N/A	N/A
OCD16	MSH6	MISSENSE	V509A	rs63751005	c.620T>C	0.001	1	DEL	Probably Damaging	HIGH	DEL	DC	5.35	DM?
OCE17-2	MUTYH	MISSENSE	Y179C	rs34612342	c.494A>G	0.002	1	DEL	Probably Damaging	VERY HIGH	DEL	DC	5.01	DM
OCQ15	MUTYH	MISSENSE	G396D	rs36053993	c.1145G>A	0.003	1	DEL	Probably Damaging	VERY HIGH	DEL	DC	5.4	DM
OCN71	NBN	MISSENSE	I171V	rs61754966	c.511A>G	0.001	1	TOL	Probably Damaging	LOW	NEUT	DC	4.81	DM
OCH30	PALB2	MISSENSE	G998E	rs45551636	c.2993G>A	0.021	2	DEL	Probably Damaging	LOW	DEL	DC	5.84	DP
OCE17-2	PALB2	MISSENSE	G998E	rs45551636	c.2993G>A	0.021	2	DEL	Probably Damaging	LOW	DEL	DC	5.84	DP
OCE17-2	PALB2	MISSENSE	E672Q	rs45532440	c.2014G>C	0.029	3	TOL	Benign	N/A	NEUT	PM	1.83	DM?
OCH26	PALB2	MISSENSE	E672Q	rs45532440	c.2014G>C	0.029	3	TOL	Benign	N/A	NEUT	PM	1.83	DM?
OCH30	PALB2	MISSENSE	E672Q	rs45532440	c.2014G>C	0.029	3	TOL	Benign	N/A	NEUT	PM	1.83	DM?
OCJ3	PALB2	MISSENSE	H1170Y	rs200283306	c.3508C>T	0.0001	1	TOL	Possibly Damaging	N/A	N/A	N/A	N/A	N/A
OCN2	PALB2	MISSENSE	L939W	rs45478192	c.2816T>G	0.0015	1	DEL	Probably Damaging	N/A	DEL	DC	5.81	DM
OCJ19	PALB2	MISSENSE	L337S	rs45494092	c.1010T>C	0.019	1	DEL	Benign	N/A	NEUT	PM	2.61	DM?
OCE17-2	PMS2	MISSENSE	N335S	rs200513014	c.1004A>G	0.0004	1	DEL	Probably Damaging	N/A	DEL	DC	5.73	DM

**2.1** Clinically Actionable Variants are those of high impact (frameshift or stop gain) in genes already associated with either breast or ovarian cancer. **2.2** Rare and predicted to be deleterious/damaging variants of unknown clinical significance in sample. AA = Amino acid change, MAF = Minor allele frequency (ExAC, European non-Finnish) OBS = Number of times variant was observed in sample. MUT PRED = Mutation predictor risk assessment, LRT = Likely hood Ratio Test for functional predicting of mutation, DEL = deleterious, TOL = Tolerated NEUT = Neutral, MUT TAST = Mutation Taster prediction, DC = Probably Disease Causing, PM = Probably Polymorphism, GERP = Genomic Evolutionary Rate Profiling, a score above 2 indicates a highly constrained sequence, HGMD Variant class; DM = Disease causing mutation, DM? = Possible disease causing mutation, DP = Disease associated mutation, N/A = Not Available. All variants listed were confirmed by Sanger DNA Sequencing.

The second gene featuring pathogenic variants in our sample, *RAD51D* (paralog of *RAD51*), has recently been identified as a moderately penetrant gene in hereditary ovarian cancer[8][9][10]. RAD51D forms a complex with RAD51B, RAD51C and XRCC2 in order to bind single stranded DNA, a necessary process for DNA repair by homologous recombination and is required for RAD51 foci formation upon DNA damage induction[11]. Although rare among familial breast cancer patients[9], loss of function variants in *RAD51D* have been associated with a relative risk of OVCA of 6.30. (95% CI 2.86–13.85)[12]. Two pathogenic nonsense SNPs in *RAD51D* were discovered in our sample. One carrier (OCH26) was diagnosed with OVCA at the age of 61, and had a family history of prostate (n = 2), breast (n = 2) and ovarian cancer on her paternal side, while the second carrier (OCK1), diagnosed at 67, had a comparatively weak family history with a single diagnosis of colon cancer on her paternal side and lung cancer in a maternal aunt.

In addition, we also identified a pathogenic nonsense mutation in a non-panel gene, *FANCM* (rs144567652, p.R1931*). This variant has been recently associated with increased risk of breast cancer (OR of 3.93)[5], warranting contact for further counseling. *FANCM* is the most highly conserved member of the Fanconi Anemia complementation group (FANCG)[13]. This group is associated with the autosomal recessive genetic disorder, Fanconi Anemia, which is characterized by genomic instability, hypersensitivity to DNA damage induced by crosslinking agents and substantial increased risk of leukemia and other cancers[14]. Other members of the FANCG complementation group include breast and ovarian cancer associated genes; *FANCO (RAD51C)*, *FANCS (BRCA1)*, *BRCA2 (FANCD1)*, *BRIP1 (FANCJ) and PALB2 (FANCN)*. *FANCM* encodes for an ATP-dependent helicase important for the resolution of DNA:RNA hybrids, thus ensuring stability with genome duplication[13]. The nonsense variant identified here has been shown to affect protein function by also inducing exon skipping[5]. The carrier (OCJ19) of *FANCM* rs144567652 was diagnosed with OVCA at 49 years of age and had a family history of breast (n = 2), multiple myeloma, leukemia, and ovarian, all on the maternal side of her family.

### Variants of unknown clinical significance detected in HBOC panel genes

**Fig 1:**
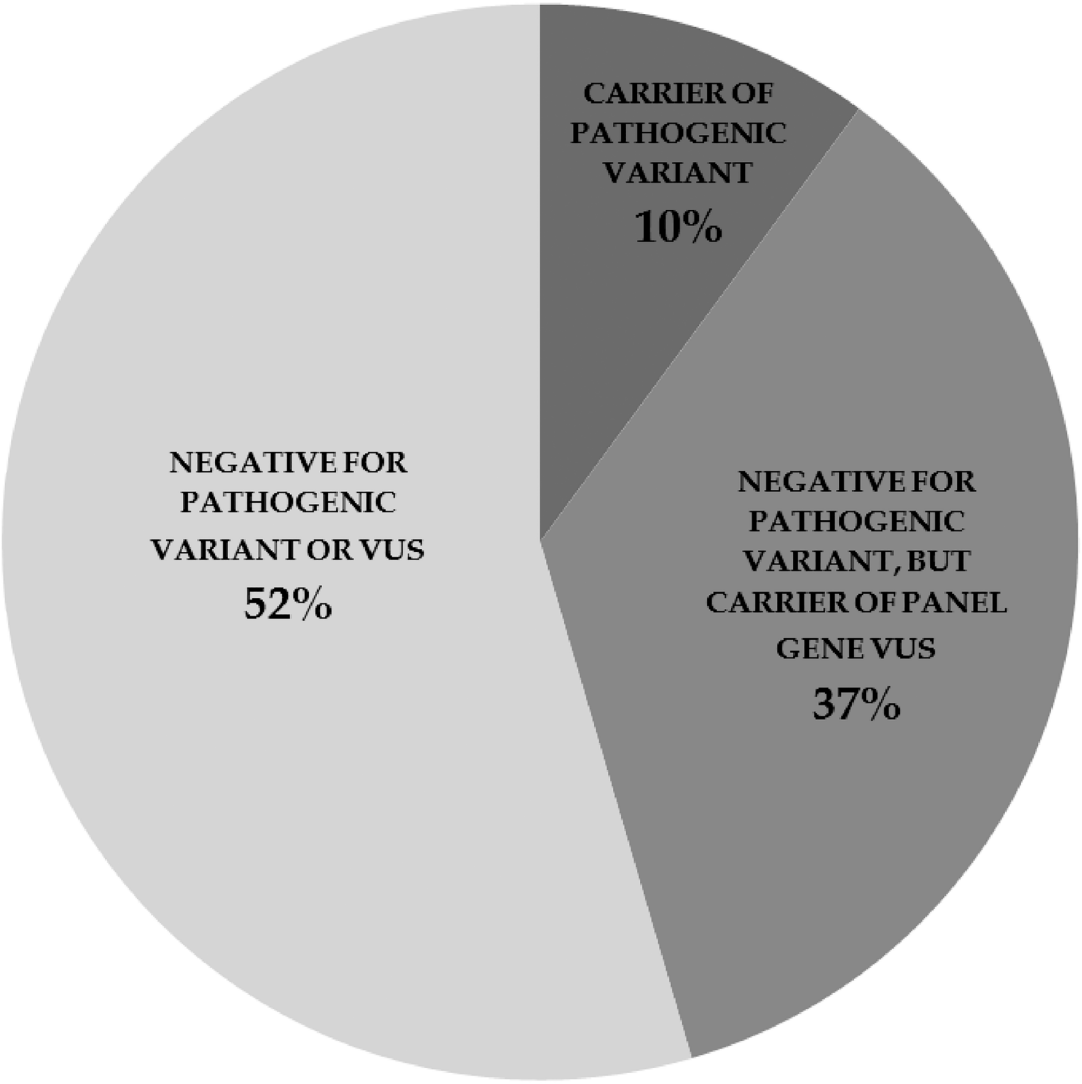
Summary of variant findings amongst our 48 subjects of high risk for genetic inheritance of OVCA. Ten percent of subjects were found to harbor a clear pathogenic variant, while 37% harbored a variant of unknown clinical significance (VUS) in a gene featured on current HBOC testing panels. 52% of subjects were found to be negative for either a clear pathogenic variant or a VUS in a known breast or ovarian cancer predisposing gene.

As most women in our sample were not found to be carriers of a clearly pathogenic mutation upon WES, we next sought to identify potentially deleterious variants in HBOC panel genes. We found that 23 women in our sample (37%) harbored one or more rare and predicted to be damaging variants of unknown significance (VUS), in panel genes; *ATM*, *BRCA1*, *BRCA2*, *CHEK2*, *MHS6*, *MUTHY*, *NBN*, *PALB2*, and *PMS2*. (Table 2.2, Fig 1) Analysis of the Human Gene Mutation Database (HGMD)[15] revealed that 6 of these variants are annotated as “disease causing” (DM), ten as “possibly disease causing” (DM?) and 3 as “disease associated polymorphism” (DP) in the Human Gene Mutation Database (HGMD)[15].

Carriers of deleterious variants in either *ATM*, *CHEK2*, *PALB2* or *NBN* are typically counseled for their risk of breast cancer, but not ovarian cancer risk despite associations in current literature[1][16][7][17]. In addition to the pathogenic *ATM* frameshift mutations discussed above, we detected an additional six rare and predicted to be damaging missense variants of unknown significance in *ATM;* rs1800054, rs138327406, rs28904919, rs1801673, rs56009889, rs35203200. The *ATM* variant rs1800054 (p.S49C) has recently been implicated as associated with a slightly increased risk for breast cancer (OR 1.08 (C.I .95–1.22) for heterozygotes, 1.44 (.39–5.32) for homozygotes [18]. *ATM* variant rs138327406 (p.F1463C MAF = 0.002) is listed as a disease causing mutation in HGMD and was found in three of six women of Ashkenazi Jewish (AJ) descent in our sample, always in combination with a second rare polymorphism 266 amino acids apart (rs2227922, p.P604S, MAF = 0.003) which was predicted to be benign. These variants were not seen in any other women in our sample and linkage data suggests they are not in disequilibrium (r^2^ = 0.5, Haploreg v4, CEU). Therefore, we suspect that there may be a founder effect resulting in the coupled segregation on a single haploblock in the AJ population. We were able to confirm in one participant (OCG29) that both variants were inherited on the same parental allele. We were not able to confirm co-segregation in the other two participants as fresh peripheral blood samples were not available to prepare RNA for this analysis. However, we did find that the unaffected daughter of OCD16 was wild type for both variants, suggesting likely co-segregation.

Similarly, with *PALB2*, we detected a pair of rare SNPs inherited together, rs45532440 (p.E672Q MAF = 0.02) and rs45551636 (p.G998E MAF = 0.02) r^2 =^ 0.69, in two unrelated individuals (OCH26 and OCE17-2). PALB2 (partner and localizer of BRCA2), physically interacts with BRCA2, is critical for the localization and stability of BRCA2 in the nucleus. Females with monoallelic germline loss of *PALB2* have a 2–4 fold increased breast cancer risk[19][20]. *CHEK2* and *NBN* are also known breast cancer associated genes that were found to each have an interesting variant of unknown significance in our sample. Female *CHEK2* and *NBN* mutation carriers are at an increased lifetime risk of developing breast cancer (2-fold for CHEK2 and 3-fold for NBN carriers)[21]. Both patients had a family history of breast cancer and the carrier of *CHEK2* had a secondary diagnosis of breast cancer herself. The p.I232V (rs587780185) variant in *CHEK2* is extremely rare (MAF = 00001) and SIFT and PolyPhen predict this alteration as deleterious and probably damaging. *NBN* p.I171V (rs61754966) has contradictory annotations among various bioinformatics assessment tools, but is called as a disease causing mutation in HGMD.

Numerous potentially deleterious VUSs in Lynch syndrome and familial adenomatous polyposis associated genes were detected in our sample. Lynch syndrome (hereditary nonpolyposis colorectal cancer), is an autosomal dominant inherited disorder caused by mutations in mismatch repair genes; *MLH1, MSH2, MSH6, PMS2*, or *EPCAM* which lead to high risk of colorectal cancer (80% lifetime risk) among others, including cancer of the ovaries (10–15% lifetime risk) and endometrium (71% lifetime risk)[22]. Skin cancer, in the form of Muir-Torre syndrome (a variant of Lynch) is another non-colonic phenotype observed in some Lynch families[23][24]. A rare (MAF = 0.007), highly conserved (GERP = 5.35) and predicted as deleterious VUS was found in the Lynch associated gene, *MHS6* (p.V509A rs63751005). The carrier of this SNP (OCD16) was diagnosed with OVCA at the age of 25, followed by a secondary diagnosis of colon cancer at the age of 65 and had a family history of colon and skin cancer as well. Two patients in our sample were heterozygous for very rare missense *MUTHY* mutations considered to be pathogenic and the cause of MYH-associated polyposis (MAP) in homozygote carriers (rs34612342 p.Y179C MAF = 0.002 and rs36053993 p.G396D MAF = 0.003). Although it is possible that a second pathogenic *MUTHY* variant occurred sporadically in the other parental allele, tissue was unavailable to detect this change in these patients. Biallelic mutations in *MUTYH* have been shown to mimic Lynch syndrome by disrupting base excision repair and resulting in a somatic loss of function of mismatch repair[25]. The carrier of the *MUTYH* variant, rs34612342, (OCE17-2) had a family history of skin and breast cancer and was a carrier of an additional VUS in the Lynch gene *PMS2* as well. The carrier of *MUTHY* rs36053993 (OCQ15) was also diagnosed with melanoma, and had a family history of colon (n = 2) skin (n = 2) and ovarian cancer.

Another conspicuous finding in our sample was the occurrence of a specific *BRCA2* truncating mutation in four unrelated individuals. The *BRCA2* variant p.K3326* (rs11571833) results in a 93 amino acid truncation and has a minor allele frequency (MAF) of 0.009 (EXAC non-Finnish). The odds ratio of observing this mutation in our sample relative to its MAF in the ExAC cohort is 4.95 (Fisher’s Exact test p-value = 0.01). It is worth noting that this allele is much more frequent in the Finnish population (MAF = .01). However, even using this more frequent MAF as our reference, our test indicates that the allele is still significantly overrepresented in our sample. p = 0.03, OR = 3.71. Although the role of *BRCA2* has been established in breast and ovarian cancer, the K3326* variant is considered to be benign by commercial testing and therefore was not identified in the initial *BRCA1/BRCA2* screening. However, recent literature is in disagreement with this classification and established that this SNP is a risk factor for lung, oral and pancreatic cancers,[26–28] all of which were observed in the family histories of the four K3326* carriers; throat (OCP36), lung (OCK1 and OCF28-1) pancreatic (OCN22), and esophageal cancer (OCN22). The accepted risk for breast cancer in carriers of this SNP is low but significant (p = 0.047, OR 1.53, 95% CI 1.00–2.34)[29]. Two of the four carriers had a family history of breast cancer, one of which had a primary diagnosis of breast cancer prior to ovarian cancer. Furthermore, analysis of the GAME-ON database[30][31], (>15000 OVCA cases and >30,000 controls) indicates that this SNP is also associated with OVCA with a p-value of 2.7x10^-4^ and OR (95% CI) = 1.31 (1.22–9.32) for all histologies, and for 8,864 invasive serous OVCA cases versus controls, the p-value was 7.11x10^-8^ and OR (95% CI) = 1.57 (1.44–1.70). This data was provided by the Ovarian Cancer Association Consortium (OCAC) (http://apps.ccge.medschl.cam.ac.uk/consortia/ocac/). These findings indicate that *BRCA2* K3326* is likely a low risk allele in ovarian cancer.

### High impact mutations in DNA repair and cell cycle control genes, not currently on HBOC testing panels

A portion of the missing heritability in OVCA is likely due to risk factors in genes not currently on testing panels. The implication of even a highly penetrant mutation would be difficult to interpret if rare, even in a mechanistically relevant gene not previously associated with the disease. Despite selecting for patients with high risk of genetic inheritance, half of the subjects in our sample were not found to harbor a pathogenic variant, nor a variant of unknown significance in any of the 24 panel genes currently tested in HBOC syndromes. (Fig 1) We therefore sought to identify rare (MAF≤0.02) mutations in our sample of high functional impact (frameshift or stop gain) in candidate genes not currently featured on testing panels. Our candidate list includes 115 genes involved DNA repair and/or cell cycle control, the two pathways most commonly associated with HBOC, in addition to 64 genes having a disease causing variant (DM) in HGMD for ovarian cancer. A full list of non- panel candidate genes analyzed is provided in supplementary information (S2 Table).

In total, 11 high impact mutations were identified in four cell cycle control genes, *CHEK1*, *RAD1*, *TP53I3* (n = 2), *MCM4*, and six DNA repair genes, *FANCM*, *HMMR*, *POLK*, *POLQ*, *RAD52* (n = 2), and *REC8* (Table 3). Importantly, this analysis resulted in the discovery of a clinically actionable pathogenic nonsense variant in *FANCM* (rs144567652) previously discussed. Most of these non-panel genes are not featured in HGMD, and are they are not analyzed during clinical testing. Therefore, we have provided the mouse phenotype seen in mouse knock-out studies when possible. A common phenotypic presentation of many known cancer predisposition genes, such as *BRCA1/2*, includes embryonic lethality in homozygote knockouts and increased cancer incidence in heterozygotes, which are reported in mouse model studies of some of these genes (Table 3).

**Table 3:** High impact mutations in DNA repair and cell cycle control genes, not Featured on HBOC testing panels.

3. High Impact Variants in DNA Repair and Cell Cycle Control Genes										
ID	Gene	Consequence	Amino Acids	dbSNP ID	Variant	MAF	OBS	Mouse Phenotype +/-	Mouse Phenotype -/-	OVARY EXPRESSION RPKM*
OCL60	CHEK1	FRAMESHIFT	G>GA Exon 7	N/A	c.1564-1565insA	N/A	1	enhanced tumorigenesis of WNT-1 transgenic mice[37]	embryonic lethal [37]	1
OCJ19	FANCM	STOP	R1931*	rs144567652	c.5713C>T	0.0009	1	none[38]	reduced life span increased cancer incidence [38]	1.1
OCK1	HMMR	STOP	E352*	rs146791423	c.1054G>T	0.0035	1	none[39]	impaired ovarian folliculogenesis[39]	.1
OCN37	MCM4	FRAMESHIFT	GGC>G Exon 12	N/A	c.1610-1611del	N/A	1	mammary adenocarcinomas in 80% of females [40]	preimplantation and embryonic lethal [40]	3.8
OCG24	POLK	FRAMESHIFT	GA>G Exon 10	N/A	c.1336del	0.0006	1	none [41]	spontaneous mutator [41]	6.2
OCG24	POLQ	STOP	Q2513*	rs148626322	c.7537C>T	0.0002	1	none [42]	increased chromosome breaks in peripheral erythrocytes [42]	8.2
OCG23	RAD1	FRAMESHIFT	CT>C Exon 6	N/A	c.1154del	N/A	1	larger, more numerous, earlier onset skin tumors with DMBA-TPA treatment [43]	embryonic lethal [43]	3.4
OCL11	RAD52	STOP	Y415*	rs4987208	c.1245T>G	0.019	1	none[44]	none[44]	7.1
OCL60	RAD52	STOP	S346*	rs4987207	c.806C>A	0.012	1			
OCL56	REC8	STOP	W365*	N/A	c.1622G>A	N/A	1	none[45]	sub-Mendelian frequencies and failure to thrive[45]	3.3
OCG14	TP53I3	STOP	S252*	rs145078765	c.755C>G	0.001	2	No knockout mouse found in the literature for this gene.	No knockout mouse found in the literature for this gene.	3.2
OCJ19	TP53I3	STOP	S252*	rs145078765	c.755C>G	0.001	2			

Rare and high impact variants (frameshift or stop gain) in sample found in DNA repair or cell cycle control genes not currently known to associate with breast or ovarian cancer. MAF = Minor Allele Frequency in Non-Finish Europeans. (ExAC). OBS = Number of times variant was observed in sample. Mouse Phenotype = Available phenotypic information on homozygote (-/-) or heterzygote (+/-) mouse knock out models. Ovary expression data RPKM (reads per kilobase per million) obtained by https://gtexportal.org.

*For reference, *OVCA genes BRCA1 =* .*6*, *BRCA2 =* .*095*, *RAD51D = 4*. All variants listed were confirmed by Sanger DNA Sequencing.

The frameshift mutation in *CHEK1* (Checkpoint Kinase 1) is notable because much like panel gene *CHEK2*, it encodes for serine/threonine protein kinase required for checkpoint-mediated cell cycle arrest and activation of DNA repair by homologous recombination repair (HRR). We also discovered a frameshift variant in *RAD1*, a gene whose protein product functions as part of the 9-1-1 cell cycle checkpoint complex to arrest cellular proliferation in the presence of incomplete DNA replication or damaged DNA, as well as in *MCM4* (Mini-chromosome maintenance complex component 4), a highly conserved helicase protein required for genome replication by initiation of replication fork formation[32]. The *TP53I3* (TP53 inducible protein 3) nonsense SNP (rs145078765 p. S252* MAF = 0.0009 is also of great interest as it was observed in two unrelated individuals in our sample. TP53I3 is an oxidoreductase-like protein and an inducer of ROS, that is transcriptionally activated by the tumor suppressor p53 and likely to be involved in p53-mediated apoptosis[33].

Among DNA repair genes, we observed high impact mutations in those encoding DNA polymerases, *POLK* (c.1336del), a translesion polymerase which initiates the continuation of replication through DNA lesions in damaged DNA, and *POLQ* (p.Q2513* rs148626322), a gene associated with micro homology-mediated end-joining pathway (MMEJ), both in a single patient. We also identified truncating variants in chromatid cohesion *REC8*, whose protein product binds sister chromatids during meiosis, and *HMMR* (hyaluronan mediated mobility receptor), which encodes for a cell motility protein that forms a complex with tumor suppressors *BRCA1* and *BRCA2*. Common missense variations in *HMMR* have been shown to modify the penetrance of breast cancer risk in *BRCA1* pathogenic mutation carriers[34]. Furthermore, we discovered two *RAD52* truncating SNPs; rs4987207 p.S346* and rs4987208 p.Y415*. *RAD52* mediates complementary ssDNA annealing and recruits RAD51 recombinase to promote recombination and homology directed DNA repair However, the *RAD*52 truncating variants that we observed in our sample have previously been found to lack an association with OVCA or breast cancer[35][36] but may modify the genetic penetrance of other variants in these pathways.

### BRCA2 K3326* truncation as a possible modifier of penetrance

**Fig 2:**
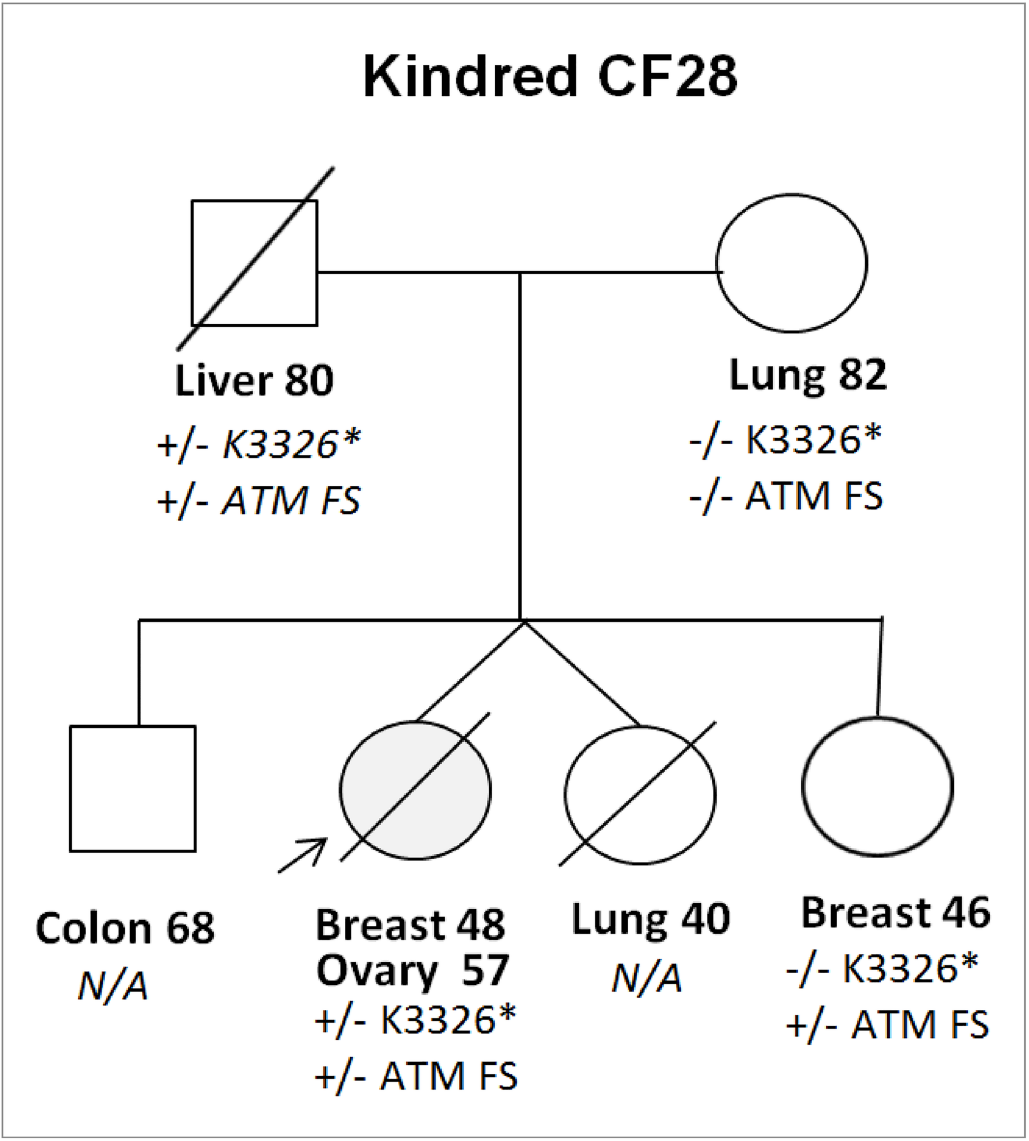
OCF28 kindred. Arrow indicates patient OCF28-1.

The prevalence of the *BRCA2* K3326* variant in our sample, along with the evidence of an association with lung, aero digestive, and pancreatic cancer [26–28] indicated that this variant may be of minimal risk when inherited alone, but may act as a modifier of penetrance to a secondary more deleterious mutation. We assert that a portion of the missing heritability in OVCA is due to this type of polygenic inheritance. This possibility led us to investigate other putative pathogenic variants that each of the four carriers had inherited in addition to *BRCA2* p.K3326* (Table 4). We therefore looked for additional rare, and moderate or high impact variants in either HBOC panel genes or candidate genes (involved in DNA repair/ cell cycle and with cancer associations in HGMD) amongst the four *BRCA2* K3326* carriers. A complete list of rare and predicted to be damaging variants of moderate impact in cell cycle and DNA repair genes is available in supporting information (S1 Table). In analyzing relevant candidate genes, we identified two patients who along with K3326* were carriers of additional, clearly pathogenic variants; a *RAD51D* nonsense mutation (OCK1) and *ATM* frameshift mutation (OCF28-1). This observation is interesting because BRCA2 interacts with the RAD51 paralogs and a *BRCA2/RAD51D* double knockdown leads to a greater loss of cellular viability [46] (and Stafford and Tainsky, preliminary data). The carrier of both the *ATM* frameshift and *BRCA2* K3326* variants developed both breast and ovarian cancer. Sequencing of some of her immediate family members at these loci determined that both variants were inherited from her father, who died of liver cancer and a twin sibling and paternal grandfather of the patient, both of which died of lung cancer a disease associated with this SNP, but whose genotypes are not available[47]. A second female sibling of this patient had inherited the *ATM* frameshift but not the *BRCA2* K3326* variant, and developed breast cancer at the age of 46 (Fig 2).

**Table 4:** BRCA2 K3326* truncation as a possible modifier of penetrance.

4. Carriers of BRCA2 K3326* and additional variants of interest									
Patient ID	Gene	Consequence	Amino Acids	dbSNP ID	Variant	MAF	HGMD cancer phenotype associated with gene	SIFT	PolyPhen
OCF28-1	ATM	FRAMESHIFT	CATCTG>C Exon 13	N/A	c.2503_2507del	N/A	Breast/Ovarian	N/A	N/A
	BRCA2	STOP	K3326*	rs11571833	c.9976A>T	0.009	Breast/Ovarian	N/A	N/A
	PALLD	MISSENSE	R303S	rs138897963	c.909A>T	0.001	Pancreatic	TOL	Probably Damaging
OCK1	ATM	MISSENSE	F1463C	rs138327406	c.4388T>G	0.002	Breast/Ovarian	DEL	Probably Damaging
	BRCA2	STOP	K3326*	rs11571833	c.9976A>T	0.009	Breast/Ovarian	N/A	N/A
	ERCC6	MISSENSE	M713V	rs201486862	c.2137A>G	0.00001	Basal cell carcinoma, Cockayne syndrome,	DEL	Benign
	HMMR	STOP	E352*	rs146791423	c.1054G>T	0.003	None	N/A	N/A
	RAD51D	STOP	R206*	rs387906843	c.616C>T	0.00001	Breast/Ovarian	N/A	N/A
	RECQL	MISSENSE	C321Y	rs150889040	c.962G>A	0.00001	Breast	N/A	Probably Damaging
OCN22	BRCA2	STOP	K3326*	rs11571833	c.9976A>T	0.009	Breast/Ovarian	N/A	N/A
	BUB1B	MISSENSE	E409D	rs28989188	c.1227A>C	0.0004	Gastrointestinal	TOL	Probably Damaging
OCP36	BRCA2	STOP	K3326*	rs11571833	c.9976A>T	0.009	Breast/Ovarian	N/A	N/A
	AXIN1	MISSENSE	V340M	rs143974067	c.1018G>A	0.00004	Colorectal adenoma	DEL	Probably Damaging

Rare and predicted to be deleterious/damaging variants (SIFT/PolyPhen-2) found in carriers of *BRCA2* p.K3326*. MAF = Minor allele frequency (ExAC, European non-Finnish) OBS = Number of times variant was observed in sample, DEL = deleterious TOL = Tolerated, N/A = Not Available. All variants listed were confirmed by Sanger DNA Sequencing.

## Discussion

We performed WES on 48 women with OVCA suspected to have inherited cancer predisposition, yet, were previously tested and found negative for known pathogenic mutations in either *BRCA1* or *BRCA2*. In doing so, we discovered pathogenic variants in *ATM* (n = 2) and *FANCM* (n = 1), genes currently associated with breast cancer but not OVCA, as well as in a gene recently implicated in hereditary ovarian cancer risk, *RAD51D* (n = 2). These findings suggest that carriers of *ATM* and *FANCM* pathogenic mutations are possibly at elevated risk of developing OVCA as well as breast cancer and that the underling genetics of these two cancers may overlap more than previously believed. Available expression data via GTEx Portal (Broad Institute) indicate both of these genes have higher RPKM (reads per kilobase per million) scores in ovary tissue versus breast; ATM = 3.6 breast, 8.7 ovary and FANCM = .89 breast, 1.1 ovary. (https://gtexportal.org) Furthermore, our findings indicate that there is incentive in resequencing *BRCA1/2* negative individuals that fit current NCCN guidelines and whose genetic risk was assessed before the era of multi-gene panel testing.

The majority of the high risk OVCA participants in our WES sample set did not harbor a clinically actionable cancer predisposing mutation upon reanalysis with whole exome sequencing, emphasizing the current challenge for genetic testing and counseling in clinical cancer care. Despite the large heritable component to OVCA, the majority of underlying genetic risk remains unexplained[30]. Although we discovered many novel putative risk loci, most are rare or private familial missense mutations of unknown clinical significance and not found in the published literature. The rarity of these variants also means that they would not be identifiable in GWAS studies. This finding is consistent with the observation that many high risk women who undergo testing for HBOC are found to be carriers of one or more “variants of unknown significance” (VUSs),[48] a rare, generally missense, mutation unannotated in its consequence to disease risk rather than a clearly pathogenic variant. Although the functional consequence of high impact variants such as nonsense and frameshift mutations are straightforward to interpret, missense mutations which result in single amino acid substitutions are not. We observed suspicious missense VUSs in HBOC panel genes employing well-accepted bioinformatic techniques: *BRCA1*, *BRCA2*, *CHEK2*, *MUTHY*, *MHS6*, *NBN*, *PMS2*, and most notably in *ATM* and *PALB2*. Overall, we found such suspicious variants in 23 of our 48 test subjects.

The ability to assess VUSs is crucial to closing the gap in unexplained heritability while aiding in more informed clinical decisions. A common approach to implicating a VUS is by linkage analysis, whereby the causal mutation is expected to segregate with the disease in one or more families. Unfortunately, DNA samples from other affected and non-affected family members are generally not often readily available, as is the case with most of the individuals in our sample. A linkage analysis is also not ideal for low to moderate risk factors because in general these variants are not highly penetrant. Bioinformatic prediction tools for variant consequence on protein function, such as SIFT and PolyPhen, are very useful for prioritizing variants for follow up. However, *in silico* assessment tools such as these often contradict each other and are not considered to have enough sensitivity and specificity to inform clinical decisions[49]. Despite the advent of detailed guidelines for variant interpretation, many variants in ClinVar list numerous testing facility submissions with conflicting interpretations of pathogenicity. Thus, the vast majority of single nucleotide polymorphisms (SNPs) in cancer-relevant genes remain unannotated as to whether the change is deleterious to protein function and potentially disease causing.

Further complicating this issue is that under a polygenic model for hereditary cancer, carriers of multiple low penetrant genetic variants could be at high risk,[50] meaning much of the unexplained heritability in OVCA may be due to more than one genetic risk factor that, when inherited together, have an additive or synergistic effect. One variant in *BRCA2* (p.K3326*) stood out as a possible modifier of penetrance due to an almost five fold increased occurrence over expected and the observation that two of the four women carrying this SNP also had a pathogenic mutation of low-to-moderate effect in a second gene involved in DNA repair, (*ATM* and *RAD51D)*. This SNP results in a 93 amino acid truncation and is reported as benign according to genetic testing services, mostly due to weak disease co-segregation in familial studies. This assessment has been questioned in recent literature due to its association with other cancers. Functional data have suggested that K3326* acts similar to wild type *BRCA2* for recombination repair and MMC sensitivity [51]. However, the K3326* truncation is located at the C-terminus of the BRCA2 protein (exon 27), and deletion of this domain has been shown to result in reduced cellular response to stalled and collapsed replication forks,[52] hypersensitivity to gamma-radiation and premature senescence[53]. Additional evidence in the literature along with our findings suggest the possibility that this variant that may be of minimal effect alone, but enhances the penetrance of another moderately damaging inherited variant in the same functional pathway. This would explain the weak genotype to phenotype correlation with this variant as well as the observation that this variant has been found in trans with other pathogenic *BRCA2* mutations, without causing Fanconi Anemia. Due to our small sample set, the occurrence of this SNP with additional moderate pathogenic mutations in the same pathway could be by chance. However, in agreement with our hypothesis of role as a possible modifier of penetrance, the *BRCA2* K3326* truncation is found in The Cancer Genome Atlas (TCGA) in the analysis of the sequence of tumor DNAs database three times, each in OVCA patients who are all also carriers of pathogenic genetic variants; (TCGA-24-1562-01 with an *NF1* frameshift, aTCGA-13-1512-01 and TCGA-23-1026-01 with *BRCA1* frameshifts, (http://cancergenome.nih.gov/).

It is likely additional risk genes exist that, when mutated, predispose to breast and/or ovarian cancer, but have yet to be implicated due to their rarity or low penetrance. In our attempt to discover novel OVCA predisposition genes, we chose to focus on genes involved in DNA repair or cell cycle control as these two dynamic and interrelated pathways are crucial to genomic stability and are the most mutated pathways in hereditary breast and ovarian cancers. In doing so, we discovered 11 high impact mutations in genes that are not featured on current HBOC risk assessment panels (*CHEK1*, *FANCM*, *HMMR*, *MCM4*, *POLK*, *POLQ*, *RAD1*, *RAD52*, *REC8*, and *TP53I3*) but have very similar or overlapping functions to those genes on commercial panels. The finding of a pathogenic variant in *FANCM* during this specific analysis is promising as it affirms our candidate gene rationale and to our knowledge, marks the first known case of a *FANCM* deleterious variant in an ovarian cancer patient. Of the eleven variants discovered in this analysis, five were novel. The rarity of these high impact variants is likely due to the essential natures of the DNA repair and cell cycle pathways. Knock out mouse model studies of *CHK1*, *MCM4*, and *RAD1* all show embryonic lethality in homozygous null mice and increased cancer incidence in heterozygotes, similarly to *BRCA1/2*, which makes them compelling and worthy of following up with functional studies. Our study is the first of its kind to describe these germline loss of function variants in ovarian cancer patients with inherited risk. Further work should include analyzing genes in other cancer related pathways since risk loci may also occur in genes not involved in DNA repair or cell cycle control.

In summary, additional WES studies on OVCA patients chosen based on family history but with no known pathogenic mutation in either *BRCA1/BRCA2* are necessary as they provide a rich resource for the discovery of novel disease risk loci. Our findings suggest that the likely sources of the unexplained etiology in OVCA is due to rare VUSs in panel genes that because of their low frequency have yet to be implicated, risk loci occurring in non-panel genes involved in DNA repair or cell cycle control, and polygenic risk inheritance. One key challenge facing genetic testing and counseling in clinical cancer care represents the functional significance of VUSs in cancer associated genes, which is necessary to provide genetics professionals with guidance for better informed patient risk evaluation, risk reduction strategies and possibly improved therapy modalities. Because a single low-to-moderately deleterious mutation may appear inconsequential alone, but could modify the penetrance of a deleterious mutation in the same pathway, combining the risk of multiple genetic variants in the presence of a second low-to-moderately deleterious mutation may also lead to better risk assessment. Therefore, definitive functional tests are necessary to discriminate variants of decreased function from benign polymorphisms and ideally, could reveal the impact of multiple low effect mutations as a means of personalized genetic risk evaluation.

## Materials and methods

### Sample ascertainment and description

Study samples were acquired through the Karmanos Cancer Institute Genetic Registry (KCIGR), an IRB approved bio specimen repository comprising females with a personal or family history of breast and/or ovarian cancer and at elevated risk of harboring a *BRCA1/2* mutation. Over 800 DNA samples from breast and/or ovarian cancer patients were collected spanning the years of 1999–2013, when HBOC genetic screening was limited to *BRCA1/2* and risk assessment was performed using BRACAPRO and Myriad II, which were the standard of care during the duration of accrual. BRCAPRO is a computer-based Bayesian probability model that uses breast and/or ovarian cancer family history to determine the probability that a *BRCA1* or *BRCA2* mutation accounts for the pattern of these cancers in the family[54]. Key attributes of consideration include the population prevalence of *BRCA* mutations, age-specific penetrance, and Ashkenazi Jewish heritage. Myriad II is a set of prevalence tables categorized by ethnic ancestry (Ashkenazi Jewish or non-Ashkenazi Jewish), breast cancer age of onset (age ≤50 years), and the presence of ovarian cancer, in the patient and/or first- or second-degree relatives. Myriad II is based on historical test data from the Myriad Genetic Laboratories clinical testing service[55].

Through the KCIGR biospecimen repository, we obtained 89 DNA samples from high risk Caucasian women with a personal history of OVCA. Participants were either confirmed *BRCA1/2* mutation carriers or *BRCA1/2* negative after full gene sequencing, BART (BRCAnalysis rearrangement test) or testing for the three common Ashkenazi Jewish mutations (Myriad Genetics Laboratories, Salt Lake City, Utah). Participants testing positive for pathogenic *BRCA1/2* germline mutations were excluded from our study sample. The final sample consists of 48 *BRCA1/2* mutation negative Caucasian OVCA patients from 47 families (one mother-daughter pair). Informed consent was signed and permission was obtained for the collection of blood samples and for access to medical records for all subjects. The protocol (HIC#024199MP2F(5R)) was approved following Full Board Review by the Human Investigation Committee at Wayne State University, Detroit, Michigan.

Information regarding tumor histology, tumor grade and age of diagnosis is summarized in Table 1. Tumor histology from study sample patients included serous (n = 26), endometrioid carcinoma (n = 5), mixed (n = 4) adenocarcinoma (n = 2), mucinous (n = 1), clear cell (n = 1), and undefined (n = 9). Tumor grades includes grade 2 (moderately differentiated, n = 6), grade 3 (poorly differentiated, n = 24), and grade 1 (well differentiated, n = 1). Ovarian cancer was the primary diagnosis for 43 patients, while four had a primary diagnosis of breast cancer and one of cervical cancer followed by a secondary OVCA diagnosis. Of those with a primary OVCA diagnosis, six had secondary cancer diagnosis: two breast, two colon, one uterine and one melanoma.

### Whole exome sequencing and candidate gene analysis

DNA from peripheral blood samples was isolated by the Karmanos Applied Genomics Technology Center, Detroit, MI using QIAamp DNA mini kit (Qiagen) and whole exome sequencing was performed using Nextera Rapid Capture Kit. Samples were demultiplexed using Illumina’s CASAVA 1.8.2 software[56]. Read quality was assessed with FastQC (http://www.bioinformatics.babraham.ac.uk/projects/fastqc/2) and alignment to the human reference genome (hg19)[57] was performed using Burrows Wheeler Aligner (BWA)[58]. PCR duplicates were removed using samtools,[59] and subsequent local realignment, Qscore recalibration, variant calling and filtering was performed using Genome Analysis Toolkit (GATK) [60] Unified Genotyper. SNPs were filtered out if: 1. Four or more alignments have mapping quality = 0 and the number of alignments that mapped ambiguously were more than 1/10 of all alignments for the given SNP2. SNP is represented by less than 5 reads 3. SNP quality is below 50 4. QD score (variant confidence) is below 1.5. Variant files were constructed using Genome Trax BIOBASE biological databases analysis software (http://www.biobase-international.com) and annotated with Illumina BaseSpace VariantStudio application v2.2.4. (www.illumina.com) and variant effects with snpEff[61].

Candidate genes analyzed for potential risk variants included those currently featured on HBOC genetic testing panels by Ambry OvaNext and Myriad MyRisk: *BRCA1*, *BRCA2*, *MLH1*, *MSH2*, *MSH6*, *PMS2*, *EPCAM*, *APC*, *MUTYH*, *CDKN2A*, *CDK4*, *TP53*, *PTEN*, *STK11*, *CDH1*, *BMPR1A*, *SMAD4*, *PALB2*, *CHEK2*, *ATM*, *NBN*, *BARD1*, *BRIP1*, *RAD51C*, *RAD51D*, in addition to 155 non-panel candidate genes important to DNA damage response or cell cycle regulation and 64 genes listed as having disease causing mutations associated with OVCA in HGMD. A full list of non- panel candidate genes analyzed is provided in supplementary information (S2 Table). In silico variant analysis of single nucleotide polymorphisms (SNPs) was performed using online available bioinformatics tools, HGMD[15], dbSNP[62], ExAC (http://exac.broadinstitute.org/), SIFT[63] and PolyPhen[64]. Variants were stringently filtered to include only exonic SNPs most likely to have a moderate to high effect impact on protein function (frameshift, nonsense, and missense) while also rare, (<0.02 MAF ExAC; European, non-Finnish) and predicted to be damaging by SIFT and/or PolyPhen, which take into consideration parameters such as amino acid substitution and evolutionary conservation. Variants passing these criteria were confirmed by forward and reverse strand Sanger sequence (Genewiz, https://www.genewiz.com) unless otherwise specified. Primers for PCR amplification and targeted sequencing were designed using Primer3Plus (http://www.bioinformatics.nl/cgi-bin/primer3plus/primer3plus.cgi). A full list of primers can be found in supplementary information (S3 Table).

### Co-segregation analysis of ATM SNPS; rs2227922 and rs138327406

Total RNA was extracted from peripheral blood lymphocytes using Qiagen RNeasy midi kit. One microgram was used as a template for a two-step RT-PCR reaction. First, a 3Kb cDNA was synthesized using *ATM* specific antisense primer; *acctgtttctgaacctccacct* and SuperScript III Reverse Transcriptase as described (Invitrogen cat# 18080–093). Next, a conventional PCR reaction was performed containing 2 μl of first strand cDNA, 10ul of 10x Qiagen master mix (cat. No.203743), 2μl of coral dye (Qiagen cat. No. 203743) and 5 pmolar ATM nested primers each: sense; *ggcactgaccaccagtatagttc* and antisense; *tggtggtgttcacattctgg*. The cycling program used to amplify the *ATM* cDNA was: 94^°^C for 2 minutes (1 cycle), 96^°^C for 5 seconds, 52^°^C for 5 seconds, 68^°^C for 1 minutes and 30 seconds (40 cycles), 72^°^C for 10 minutes. The 3kb band obtained was cloned in TOPO vector (TOPO TA cloning kit Invitrogen cat. # 45–0641) and 2 μl of the reaction was transformed in stellar competent cells (Clontech cat. No. 636763). Twelve clones were amplified and sent to Genewiz for sequencing, using MP3 sense and antisense primers provided by the TOPO TA kit.

## Supporting information

S1 Table Rare missense variants in non-HBOC panel genes involved in DNA repair or cell cycle control and are associated with cancer phenotypes in HGMD. MAF = Minor Allele Frequency in (ExAC Non-Finnish Europeans.). DEL = Deleterious TOL = Tolerated, N/A = Information not available, *Variants in these genes were not confirmed by Sanger DNA sequencing.
(DOCX)

S2 Table Full list of non-panel genes analyzed, involved in either DNA repair cell cycle control, or listed as having “disease causing mutations” (DM) associated with OVCA in HGMD. (DOCX)

S3 Table Primer sequences. (DOCX)

S1 Text VCF files available at figshare; https://figshare.com/s/7471a180cd770aeda2fd. (DOCX)
