# Supplementary material for: Reanalysis of BRCA1/2 negative high risk ovarian cancer patients reveals novel germline risk loci and insights into missing heritability
Source: PLoS One. 2017 Jun 7;12(6):e0178450. doi: 10.1371/journal.pone.0178450 (PMC5462348; doi:10.1371/journal.pone.0178450)
Supplement: S1 Table — MAF = Minor Allele Frequency in (ExAC Non-Finnish Europeans.). DEL = Deleterious TOL = Tolerated, N/A = Information not available, *Variants in these genes were not confirmed by Sanger DNA sequencing. (DOCX) [file pone.0178450.s001.docx]

| **S1 Missense Variants in Non HBOC Panel DNA Repair and Cell Cycle Control Genes that are Associated with Cancer Phenotypes in HGMD** | | | | | | | |
| --- | --- | --- | --- | --- | --- | --- | --- |
| **Gene** | **Amino Acids** | **dbSNP ID** | **MAF** | **OBS** | **HGMD (cancer phenotype associated with gene)** | **SIFT** | **Polyphen** |
| APEX1* | P248L | rs201100630 | 0.00005 | 1 | Head and Neck | DEL | Benign |
| AXIN1 | V340M | rs143974067 | 0.00004 | 1 | Colorectal adenoma | DEL | Probably Damaging |
| BUB1B | E409D | rs28989188 | 0.0004 | 1 | Gastrointestinal | TOL | Probably Damaging |
| CASP10* | I406L | rs80358239 | 0.004 | 1 | Autoimmune lymphoproliferative syndrome II | TOL | Possibly Damaging |
| ERCC4* | E875G | rs1800124 | 0.019 | 1 | Lung, Cockayne, Xeroderma pigmentosa, Breast/Ovarian, Fanconi anaemia, | DEL | Possibly Damaging |
| ERCC6 | M713V | rs201486862 | 0.00006 | 1 | Cockayne syndrome, basal cell carcinoma, | DEL | Benign |
| EXO1* | D270V | rs201509012 | 0.0005 | 1 | Colorectal | DEL | Possibly Damaging |
| EXO1* | G759E | rs4150001 | 0.009 | 1 | Colorectal | TOL | Benign |
| FANCA* | T475M | N/A | N/A | 1 | Fanconi Anemia | DEL | Possibly Damaging |
| FANCA* | A602G | N/A | N/A | 1 | Fanconi Anemia | DEL | Possibly Damaging |
| FANCF* | P320L | rs45451294 | 0.017 | 2 | Fanconi Anemia | TOL | Probably Damaging |
| MLH3* | V741F | rs28756990 | 0.006 | 1 | Colorectal, Breast/Ovarian, | TOL | Possibly Damaging |
| PALLD* | R303S | rs138897963 | 0.001 | 1 | Pancreatic | TOL | Probably Damaging |
| PMS1* | T75I | rs61756360 | 0.0008 | 1 | Breast/ovarian | DEL | Probably Damaging |
| RAD50* | T191I | rs2230017 | 0.0007 | 1 | Breast/Ovarian | DEL | Benign |
| RBL1* | R199H | N/A | N/A | 1 | Multiple adenoma | DEL | Probably Damaging |
| RBL1* | E624Q | N/A | N/A | 1 | Multiple adenoma | TOL | Possibly Damaging |
| RECQL | C321Y | rs150889040 | 0.00001 | 1 | Breast | N/A | N/A |
| WRN* | T573A | rs150148567 | 0.001 | 1 | Colorectal, Breast, Pancreatic, Werner syndrome | DEL | Probably Damaging |

**Table S1** Rare missense variants in non-HBOC panel genes involved in DNA repair or cell cycle control and are associated with cancer phenotypes in HGMD. MAF=Minor Allele Frequency in (ExAC Non-Finnish Europeans.). DEL= Deleterious TOL= Tolerated, N/A= Information not available, *Variants in these genes were not confirmed by Sanger DNA sequencing.
