# Supplementary material for: Reanalysis of BRCA1/2 negative high risk ovarian cancer patients reveals novel germline risk loci and insights into missing heritability
Source: PLoS One. 2017 Jun 7;12(6):e0178450. doi: 10.1371/journal.pone.0178450 (PMC5462348; doi:10.1371/journal.pone.0178450)
Supplement: S2 Table — (DOCX) [file pone.0178450.s002.docx]

**S2 Table. Full list of Non-Panel genes analyzed, involved in either DNA repair cell cycle control, or listed as having “disease causing mutations” (DM) associated with OVCA in HGMD.**

**DNA repair cell cycle control genes:**

*APEX1*

*APEX2*

*BLM*

*DDB1*

*DDB2*

*ERCC1*

*ERCC2*

*ERCC3*

*ERCC4*

*ERCC5*

*ERCC6*

*ERCC8*

*EXO1*

*FANCA*

*FANCC*

*FANCE*

*FANCF*

*FANCG*

*FEN1*

*LIG1*

*LIG3*

*LIG4*

*MBD4*

*MGMT*

*MLH1*

*MLH3*

*MPG*

*MSH2*

*MSH3*

*MSH5*

*NEIL1*

*NTHL1*

*OGG1*

*PARP1*

*PCNA*

*PMS1*

*POLB*

*POLD1*

*POLD2*

*POLD3*

*POLD4*

*POLE*

*POLE2*

*POLE3*

*RAD1*

*RAD17*

*RAD50*

*RAD51*

*RAD51C*

*RAD52*

*RECQL*

*RECQL4*

*RECQL5*

*RFC1*

*RFC2*

*RFC3*

*RFC4*

*RFC5*

*RPA1*

*RPA2*

*RPA3*

*SMUG1*

*TDG*

*UNG*

*UNG2*

*WRN*

*XPA*

*XPC*

*XRCC1*

*XRCC2*

*XRCC3*

*XRCC4*

*XRCC5*

*XRCC6*

*ZFP276*

*FANCM*

*HMMR*

*POLK*

*POLQ*

*REC8*

*ABL1*

*ANAPC2*

*ANAPC4*

*ATR*

*BAX*

*BCCIP*

*BCL2*

*BIRC5*

*CCNB1*

*CCNB2*

*CCNC*

*CCND1*

*CCND2*

*CCNE1*

*CCNF*

*CCNG1*

*CCNG2*

*CCNH*

*CCNT1*

*CCNT2*

*CDC16*

*CDC2*

*CDC20*

*CDC34*

*CDK2*

*CDK5R1*

*CDK5RAP1*

*CDK6*

*CDK7*

*CDK8*

*CDKN1A*

*CDKN1B*

*CDKN2A*

*CDKN2B*

*CDKN3*

*CHEK1*

*CKS1B*

*CKS2*

*CUL1*

*CUL2*

*CUL3*

*DDX11*

*DIRAS3*

*DNM2*

*E2F4*

*GADD45A*

*GTF2H1*

*GTSE1*

*HERC5*

*HUS1*

*KNTC1*

*KPNA2*

*MAD2L1*

*MAD2L2*

*MCM2*

*MCM3*

*MCM4*

*MCM5*

*MKI67*

*MNAT1*

*RAD9A*

*RB1*

*RBBP8*

*RBL1*

*RBL2*

*SERTAD1*

*SKP2*

*SKP2.*

*SUMO1*

*TFDP1*

*TFDP2*

*UBA1*

*TP53I3*

*TP53BP1*

*TP53BP2*

**Genes listed as having association with OVCA in HGMD:**

*ACACA*

*ADH1B*

*AGER*

*AGO2*

*APOBEC3B*

*ARL11*

*ATAD5*

*ATF1*

*BPIFC*

*CAV1*

*CLTC*

*COL18A1*

*CREBBP*

*CYP17A1*

*CYP1A1*

*DCC*

*DCP1B*

*DROSHA*

*E2F2*

*EPHX1*

*FMR1*

*FRG1*

*FSHR*

*GSTM1*

*GSTO2*

*GSTP1*

*GSTT1*

*HFE*

*HIP1*

*HRAS*

*IGF2*

*IL1A*

*IL23R*

*ILI6*

*ILIR1*

*ITK*

*KI*

*KMT5A*

*KRAS*

*LIN28B*

*LPAR6*

*LPL*

*MIR191*

*MIR423*

*MTR*

*MYH9*

*NCKIPSD*

*NEKB1*

*NOTCH*

*OGG1*

*P14ARF*

*PCM*

*PGR*

*PPMID*

*PSMC3IP*

*RB1*

*RNF213*

*SLC4A7*

*SLX4*

*SPINK1*

*TERT*

*TNFRSF13B*

*UGT2A3*

*WAS*
