# Supplementary material for: Reanalysis of BRCA1/2 negative high risk ovarian cancer patients reveals novel germline risk loci and insights into missing heritability
Source: PLoS One. 2017 Jun 7;12(6):e0178450. doi: 10.1371/journal.pone.0178450 (PMC5462348; doi:10.1371/journal.pone.0178450)
Supplement: S3 Table — (DOCX) [file pone.0178450.s003.docx]

**S3 Table. Primer Sequences**

| **Gene** | **dbSNP ID** | **Variant** | **Forward Sequencing Primer** | **Reverse Sequencing Primer** |
| --- | --- | --- | --- | --- |
| ATM | N/A | c.2503_2507del | AAGAAGAACTTTCATTCTCAGAAGTAG | TTTTTCATCATATAATCCCTATGCTC |
| ATM | N/A | c.5697_5698insA | TGGTGTACTTGATAGGCATTTGA | TCTGAGCTTTTCCACACTGC |
| ATM | rs1800054 | c.146C>G | GAGCTACAGAACGAAAGGTAGTAAA | TTTCCTCTAATCTGAAGTCTTGTGAA |
| ATM | rs138327406 | c.4388T>G | ATCAGAAAATTCTTCTTGCCATA | CAGGAGGTTGAGGATGCAGT |
| ATM | rs28904919 | c.998C>T | TTTTGTGGGAGCTAGCAGTG | GGTGGCTCATGCCTGTAATC |
| ATM | rs56009889 | c.6919C>T | GTGGGGAGATGTCATGCAG | GGGACACCAATGCCTCTACTT |
| ATM | rs35203200 | c.7618G>A | CCTCAGATAAGAAAAGA | TGCAGTGGGTAGAGCGTG |
| AXIN1 | rs143974067 | c.1018G>A | TTCCTGAAGACAAAGCCCAG | GTCACTAACATGCCCTGCTT |
| BRCA1 | rs4986852 | c.3119G>A | TCCCATAGGCTGTTCTAAGTTATCTG | CAGAGGCAACGAAACTGGACTCA |
| BRCA1 | rs1800744 | c.4535G>T | TTGAGCTATTTTTCTAAAGTGGGCTTA | AGGCAACATGAATCCAGACTTCTAG |
| BRCA2 | rs80358479 | c.1889C>T | GCCTCTGAAAGTGGACTGGA | GCAGGCATGACAGAGAATCA |
| BRCA2 | rs28897747 | c.8149G>T | TAAAACTAGTAGTGCAGATACCCAAAAAGTG | CAATGACTGATTTTTACCAAGAGTGCAAA |
| BRCA2 | rs11571747 | c.8567A>C | TGGAGGAAATGTTGGTTGTGTTGA | CCTTCATGTTCTTCAAATTCCTCCTGA |
| BRCA2 | rs11571833 | c.9976A>T | CACCTGTCTCAGCCCAGATGAC | AGTTGTAATTGTGTCCTGCTT |
| BUB1B | rs28989188 | c.1227A>C | TTCCCCACTTTACGCTTTTG | ACCATAGAAGGCAGCAGTGG |
| CHEK1 | N/A | c.1564-1565insA | TGAAGTGCCTCTAAAGTTTCCA | TGTTCACACAATGATGAAACCA |
| CHEK2 | rs587780185 | c.565A>G | ATCACAGTGGCAATGGAACC | CTCCCAAAGTGCTGGGATTA |
| ERCC6 | rs201486862 | c.2137A>G | TCGGATCATTCTGTCTGGCT | ATGAGCCTGGCCATCTTTCT |
| FANCM | rs144567652 | c.5713C>T | TCTAGCACTTCAGGGGCATC | TGAAGTGAGCTGTTAGCCATCC |
| FANCM | rs144567652 | c.5713C>T | TCTAGCACTTCAGGGGCATC | TGAAGTGAGCTGTTAGCCATCC |
| HMMR | rs146791423 | c.1054G>T | ACCTCACAATGCCATTCCA | AAGCTGAAAGGCTGGTCAAG |
| MCM4 | N/A | c.1610-1611del | GCGGGACAAGGAAGGATTTT | CATGTTCACGGTGGAGAAGG |
| MSH6 | rs63751005 | c.620T>C | TGAACTGGGGCTGGTATTCA | AAGCACACACCATATGCACG |
| MUTYH | rs34612342 | c.494A>G | GTCTCTTTCTGCCTGCCTGT | CTACGTTGCCATCCACCAC |
| MUTYH | rs36053993 | c.1145G>A | AACACTGGACAGTGCCACCT | AAGGGTCAAGGGGTTCAAAT |
| NBN | rs61754966 | c.511A>G | CAACAAAGAAATTTGGGGAAC | GCAGTGACCAAAGACCGACT |
| PALB2 | rs45551636 | c.2993G>A | TTTGGCTTAGGGCATTGTTT | GACATGTCTGGCTTCCACCT |
| PALB2 | rs45532440 | c.2014G>C | CCTGATGAAGACTTTGGACCTC | TAAGATGGGGAAAGCAGGTG |
| PALB2 | rs200283306 | c.3508C>T | TCTGTCTGGACATAAACAAGCAA | ACTCTCAGCGTGGGTGTGAT |
| PALB2 | rs45478192 | c.2816T>G | ATCTTTCAGATTCTTTCAAGACTCAAGCC | CTGGATTAAACAAAAATGAAACAACCAAGC |
| PALB2 | rs45494092 | c.1010T>C | ATTTCACCAGGGCGACTACA | TTGACTCAAAGGGCTCCACT |
| PALLD | rs138897963 | c.909A>T | ACCTCAGCAGATGTTGTGTC | ATGGGTGCCTAAATGTCGGA |
| PMS2 | rs200513014 | c.1004A>G | CAGTGGCTGCTGACTGACAT | GTTGCAGTGAGCTGAGATCG |
| POLK | N/A | c.1336del | TGAATAGGCTATGGGAGAAAGAA | GGCATTTATTGCAGGGAGTG |
| POLQ | rs148626322 | c.7537C>T | TCCCAAAGAGGGTTACAGGA | AGGCTGAGCGTCAAGCTATC |
| RAD1 | N/A | c.1154del | CGGCCACCTTTAGACTCTTG | TTGGGAGTTCTGAGCAGTGTT |
| RAD51D | rs587781756 | c.511C>T | CCTGCAGCAAAACGTCCTAT | AGTAGGACACCTGCCCACAG |
| RAD51D | rs387906843 | c.616C>T | ACCACTGTGACAACTGACCA | AGTAGGACACCTGCCCACAG |
| RAD52 | rs4987208 | c.1245T>G | TAGCAGGAAGCGGAAACG | ACTGCAGTGGGCTCTCAGTC |
| RAD52 | rs4987207 | c.806C>A | TCCAGTTCCTCTTTGGTCCT | AGGATCTCCCCTTAATTTTTGTG |
| REC8 | N/A | c.1622G>A | GACCTTCCCCCACTACACAG | TGGGGATGGGAGAAGTAGAA |
| RECQL | rs150889040 | c.962G>A | GAAGCTCTGACCATCCCTGA | CAACAGTTGCCACTACTACCTG |
| TP53I3 | rs145078765 | c.755C>G | TCTGAAATCGGGTTCCCTCT | AGGCCTCATAAATGGTGAACTT |
