# Supplementary material for: Reanalysis of BRCA1/2 negative high risk ovarian cancer patients reveals novel germline risk loci and insights into missing heritability
Source: PLoS One. 2017 Jun 7;12(6):e0178450. doi: 10.1371/journal.pone.0178450 (PMC5462348; doi:10.1371/journal.pone.0178450)
Supplement: S1 Text — (DOCX) [file pone.0178450.s004.docx]

VCF files available at Figshare; https://figshare.com/s/7471a180cd770aeda2fd
